# Effective Immune Functions of Micronutrients against SARS-CoV-2

**DOI:** 10.3390/nu12102992

**Published:** 2020-09-29

**Authors:** Kashaf Junaid, Hasan Ejaz, Abualgasim Elgaili Abdalla, Khalid O. A. Abosalif, Muhammad Ikram Ullah, Humaira Yasmeen, Sonia Younas, Sanaa S. M. Hamam, Abdul Rehman

**Affiliations:** 1 Department of Clinical Laboratory Sciences, College of Applied Medical Sciences, Jouf University, Sakaka 72388, Al Jouf, Saudi Arabia; hetariq@ju.edu.sa (H.E.); aealseddig@ju.edu.sa (A.E.A.); koabosalif@ju.edu.sa (K.O.A.A.); mikramullah@ju.edu.sa (M.I.U.); 2 Department of Medical Microbiology, Faculty of Medical Laboratory Sciences, Omdurman Islamic University, Omdurman 14415, Sudan; 3 Department of Microbiology and Molecular Genetics, The Women University Multan, Multan 60000, Pakistan; humaira.6127@wum.edu.pk; 4 Department of Pathology, Tehsil Headquarter Hospital Kamoke, District Gujranwala, Kamoke 50661, Pakistan; soniamicro02@gmail.com; 5 Department of Medical Microbiology and Immunology, Faculty of Medicine, Menoufia University, Shebin El-koom 32511, Egypt; sanaa_mohamed28@yahoo.com; 6 Department of Microbiology, King Abdulaziz Specialist Hospital, Sakaka 72341, Saudi Arabia; 7 Department of Microbiology and Molecular Genetics, University of the Punjab, Lahore 54590, Pakistan; rehman_mmg@yahoo.com

**Keywords:** COVID-19, nutrients, immunonutrition, public health, immune enhancement, minerals, vitamins

## Abstract

The third coronavirus outbreak in the last two decades has caused significant damage to the world’s economy and community health. The highly contagious COVID-19 infection has affected millions of people to date and has led to hundreds of thousands of deaths worldwide. Aside from the highly infectious nature of SARS-CoV-2, the lack of a treatment or vaccine has been the main reason for its spread. Thus, it has become necessary to find alternative methods for controlling SARS-CoV-2. For the present review, we conducted an online search for different available nutrition-based therapies for previously known coronavirus infections and RNA-based virus infections as well as general antiviral therapies. These treatments have promise for combating COVID-19, as various nutrients and minerals play direct and indirect roles in the control and prevention of this newly emerged viral infection. The patients’ nutritional status with COVID-19 must be analyzed before administering any treatment, and nutritional supplements should be given to the affected individuals along with routine treatment. We suggest a potential interventional role of nutrients to strengthen the immune system against the emerging infection caused by COVID-19.

## 1. Introduction

Severe acute respiratory syndrome coronavirus 2 (SARS-CoV-2), which belongs to the family of coronaviruses (CoVs), is the causative agent of pandemic coronavirus disease 2019 (COVID-19). There are four subfamilies of *Coronaviridae*, including the α, β, γ, and δ coronaviruses [1]. SARS-CoV-2 is a β-coronavirus and has a positive-sense single-stranded RNA genome [2]. CoVs can cross the species barrier and jump from mammals and birds to humans. The previous outbreaks of CoVs include those that caused the Middle East respiratory syndrome (2012) and severe acute respiratory syndrome (2002), known as MERS and SARS, respectively [3]. In December 2019, a flulike novel coronavirus, named SARS-CoV-2 and later found to be closely related to the MERS and SARS CoVs, emerged in Wuhan, China [4]. The World Health Organization (WHO) declared COVID-19 to be pandemic a month after its first outbreak because of the extremely high morbidity and mortality rates worldwide.

Presently, most of the drugs and vaccines for treating and preventing COVID-19 are still under trial, while the number of infected individuals continues to rise. There is no definite therapy for this novel infection, making it necessary to identify other options to control and prevent the escalating number of cases. An alternative approach that may help to combat this virus is to optimize the immune system. Many scientists have stressed the importance of nutritional intervention to improve the immune response. A well-balanced diet is essential for maintaining immune homeostasis. Any micronutrient deficiency may hinder the immune response against pathogens [5]. The ability of nutrients to protect against many infectious diseases and their role in reducing lung damage in pulmonary infections have been established in recent studies [6,7].

This review emphasizes micronutrients’ role in the development and efficient functioning of the immune system, primarily the antiviral defense system. We carried out an online search in PubMed for this review using the keywords coronaviruses, MERS, SARS, nutrients, and minerals. We included a total of 107 online studies, and none of the studies were used in our work other than accessible online. We summarized the general findings on the inhibitory actions of certain minerals and vitamins and proposed guidelines that can be used for COVID-19 prevention and therapy. We anticipate that these nutrients will aid in combating the newly emerging viral infection [8].

## 2. COVID-19 and the Immune Response

Based on the clinical manifestations, patients with COVID-19 are divided into three groups, presymptomatic, asymptomatic, and symptomatic [9]. The reasons why some develop severe disease and others do not are not entirely known; however, the immune system of an infected individual is one of the primary factors. When a virus infects an individual, a specific immune response is triggered, which is essential to eradicate the virus and prevent its progression. If the host immune system is weak, the virus will propagate and cause extensive tissue damage, especially in organs that express angiotensin-converting enzyme 2 (ACE2) receptors [10].

For the virus to enter host cells, the receptor-binding domain (RBD) of the viral spike protein binds to ACE2 receptors on host cells [10,11]. The virus can be taken up by antigen-presenting cells (APCs) such as dendritic cells and macrophages, which present it to the T cells. APCs contribute to the activation and differentiation of T cells and later to the massive release of cytokines. The host’s natural immune system recognizes viral components through pattern recognition receptors (PRRs) such as toll-like receptors (TLRs). Binding to TLRs induces the expression of inflammatory factors that can mediate lung inflammation and fibrosis [12,13].

**Figure 1:**
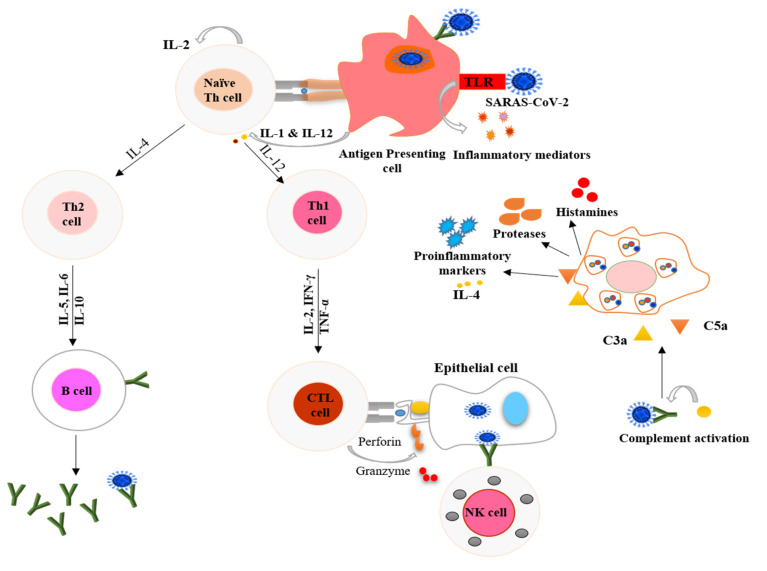
Schematic representation of processes used by the host immune system to combat COVID-19. The antigen-presenting cells (dendritic cells and macrophages) present the virus to naïve Th cells and stimulate the adaptive immune system. T cells also signal monocytes and macrophages to attract them to the infection site, where they release chemokines. Toll-like receptors (TLR) present on antigen-presenting cells recognize specific receptors on the virus and secrete inflammatory markers in innate immune system response. Mast cells activated by the pathogen release proinflammatory markers, proteases, and histamines. The upregulated secretion of proinflammatory markers causes a cytokine storm. IL-1 = interleukin 1; IL-2 = interleukin 2; IL-12 = interleukin 12; IL-4 = interleukin 4; IL-5 = interleukin 5; IL-6 = interleukin 6; IL-10 = interleukin 10, IFN-γ = interferon gamma; TNF-α = tumor necrosis factor alpha; CTL = cytotoxic T lymphocytes; NK = natural killer; C3a = complement component 3a; and C5a = complement component 5a.

Mast cells serve as a protective barrier against pathogens, and they also can be activated by a viral infection. Activated mast cells release histamines and proteases and trigger the release of proinflammatory markers, including IL-1, IL-6, and IL-33 [14]. These events lead to the activation of T cells and the massive release of cytokines, contributing to the amplification of the immune response. CD4+ or helper T cells stimulate the synthesis of virus-specific antibodies by activating B cells, whereas the function of CD8+ cells or cytotoxic T cells is to kill the virus-infected cells [12]. T helper cells also release chemokines and cytokines and provide signals to help monocytes and neutrophils reach the infected site, as shown in Figure 1 [15,16]. The extensive release of proinflammatory cytokines is known as a cytokine storm, leading to acute respiratory distress syndrome (ARDS) in these patients [17]. A primary cause of the high fatality rate of COVID-19 is the development of ARDS. The immediate release of free radicals and cytokines significantly increases oxidative stress, which is a hallmark of ARDS, causing cellular injury, multiple organ failure, and eventually death.

## 3. Nutritional Interventions for Treatment of COVID-19

Healthy and balanced nutrition is linked with strong immunity, and it represents our most potent tool in the ongoing COVID-19 crisis. Generally, poorly nourished individuals are at a higher risk of developing various types of infections [18]. Moreover, chronic and severe infections can cause nutritional disorders and worsen a patient’s nutritional status, making them susceptible to other infections. Thus, especially during the COVID-19 pandemic, it is imperative for everyone to monitor their diet and nutritional status [19]. Recent reports have identified certain groups at higher risk of COVID-19-associated complications, with the elderly and individuals with comorbidities such as hypertension, diabetes, and cancer more severely affected [20,21,22]. These risk factors are associated with malnutrition, which may alter the health status of the individuals.

In the absence of a specific antiviral therapy for SARS-CoV-2, several supportive and adjunct treatments are recommended. These include corticosteroid, ascorbic acid, anti-inflammatory, and interleukin-directed therapies. The overall aim is to manage the cytokine storm and the progression of infection [23]. The limited number of studies on the supportive care management of COVID-19 cases state that nutritional status should be assessed in all patients at the time of hospital admission. It is recommended that nutritional support be given to those in the high-risk group, asymptomatic carriers, and patients with moderate or severe COVID disease [24]. A variety of micronutrients strategies to treat COVID-19 have reached the clinical trial stage (Table 1). The immediate supplementation of certain nutrients in mild cases can prevent the progression of diseases.

Micronutrient deficiencies suppress the immune system by altering the T cell- and antibody-mediated immune response and dysregulating the host immune system [25]. A balanced diet includes healthy portions of vegetables, fruits, nuts, legumes, whole grains, and moderate levels of dairy, fish, and poultry. It is recommended to limit the intake of sugar, refined carbohydrates, processed foods, and red meat. The fats consumed should include olive oils [26,27]. The rationale behind such a diet is that it will provide the necessary amount of healthy macronutrients, essential vitamins, and minerals and ensure an excellent metabolic state and maintain a healthy body weight [28]. Healthy eating provides necessary vitamins and minerals that produce sufficient numbers of immune cells and antibodies, resulting in better immunity, which prepares the body to fight off infections.

## 4. Minerals and Immune System

### 4.1. Zinc

Zinc is a necessary mineral found in various fruits and vegetables. It plays a vital role in the maintenance and growth of adaptive and innate immune cells [29]. Zinc deficiency results in immune system dysfunction, which increases the susceptibility to infections and diseases [30]. Various studies have highlighted the role of zinc in preventing respiratory tract infections. It has been documented that zinc supplementation in children infected with measles who were zinc deficient reduced the morbidity and mortality associated with lower respiratory tract infections [31]. Recent studies have shown that increasing the concentration of intracellular zinc markedly reduces the replication of different types of RNA viruses [32]. Zinc supplementation in cases of hepatitis C and human papillomavirus infection has been shown to result in clinical improvement [33]. It has been reported that an amalgamation of low concentration zinc and zinc-ionophores retards the replication of SARS-CoVs in vitro [34].

Another approach to the treatment of COVID-19 infection is to target ACE2 receptors. SARS-CoV-2 requires ACE2 receptors to enter host cells; therefore, it is thought that these receptors are potential therapeutic sites [35]. Speth et al. found that the activity of recombinant human ACE2 in rat lungs was reduced when exposed to zinc (100 µM) [36,37]. In a recent case study, significant improvement was observed in four confirmed COVID-19 patients when treated with a high dose of zinc salt [38]. The use of zinc supplements may help to relieve COVID-19-related symptoms. 

The primary defensive mechanism by which zinc protects against bacterial and viral infections is its role as an antioxidant. Adequate zinc levels in the body protect against oxidative stress caused by reactive oxygen species (ROS) [39]. Zinc deficiency promotes the production of proinflammatory cytokines and is linked with the inflammatory alteration of lung predispose to fibrosis [40]. Supplementation of a moderate dose of zinc has been shown to correct the overproduction of proinflammatory cytokines in the elderly caused by zinc deficiency [41]. It also stimulates the proliferation of immune cells that function to remove foreign entities and prevent infections. Zinc also plays a structural role in the body in maintaining the integrity of membranes and the skin [42]. It mediates the development and function of immune cells. Zinc is involved in adaptive immunity by participating in the T lymphocytes’ development by promoting the binding of certain regulatory enzymes to T cells [39].

### 4.2. Iron

Iron is a necessary nutrient and contributes substantially to the development of immunity [43]. Iron deficiency can impair host immunity, as various immune system cells need iron for their growth and development. However, higher doses of iron can also lead to oxidative stress, creating a suitable environment for inducing harmful viral mutations [44,45]. In the context of the current COVID-19 pandemic, maintaining sufficient levels of iron could be useful, as iron deficiency can increase the risk of recurring acute respiratory tract infections [46]. Iron contributes to the upregulation of the immune response but dysregulates iron homeostasis that regulates proinflammatory cytokine production. It can mediate the synthesis of enzymes crucial for immune cell generation, including ribonucleotide reductase, which carries out DNA synthesis. Iron can generate hydroxyl radicals, which help neutrophils to kill viruses and bacteria [42]. It stimulates the synthesis of ROS for destroying pathogens and inducing the differentiation of T lymphocytes [47].

A retrospective analysis in the COVID-19 hospitalized patients indicates that most patients suffering from functional iron deficiency are significantly linked with progressive inflammation and prolonged hospital stay [48].

### 4.3. Selenium

Selenium (Se) is an important element that is required only in trace amounts in the body. Selenium content present in food products based on the amount of selenium existing in the soil. So, the amount in similar food item varies in different geographical locations. However, the primary sources for this element are meat, bread, mushrooms, dairy products, fish, seafood, and nuts [49,50,51]. It acts as an enzyme cofactor that takes part in mammalian redox reactions [52]. Because the host’s nutritional condition is crucial in protecting against various diseases [53], deficiencies in various nutrients influence the immune reaction and the viral infection [54].

A selenium-deficient diet can lead to increased oxidative stress in the body, resulting in mutating the viral genome and possibly transforming a benign virus into a highly virulent and more infectious pathogen [55]. Low selenium levels have been shown to increase the viral pathogenicity influenza virus and coxsackievirus and transform their genome from a nonvirulent to a virulent virus [56]. These genomic mutations alter the functioning of certain enzymes that regulate the levels of oxidative stress in the body, ultimately leading to tissue and organ damage [57,58].

Selenium, along with ginseng saponins, has been reported to lead to the upregulation of the reaction to a live infectious coronavirus vaccine against avian coronavirus (IBV) in chickens [59]. A recent study showed that selenium plays a particular role in ACE inhibition, suggesting its beneficial role against COVID-19 [60]. In another study, it is suggested that the preinfectious level of zinc and selenium may be of a particular significance in terms of resistance to COVID-19 progression [61]. Such findings indicate that the use of selenium supplements can help in the treatment of COVID-19. The immune effect of selenium is attributed to its role in selenoproteins (selenium-dependent enzymes) that regulate the level of oxidative stress in the body [47]. The selenoproteins mediate the antioxidant defense system that controls the functioning of leukocytes. It is also involved in T lymphocyte proliferation and production of the immunoglobulins that protect the body against infections and diseases [62].

## 5. Vitamins and Immune System

### 5.1. Vitamin A

Vitamin A is present in the body in three active forms, retinoic acid, retinol, and retinal. It is a fundamental vitamin that is fat-soluble. Because of its widely known role in the immune system and its effectiveness against infections, vitamin A is considered an “anti-infective” vitamin [63]. The appropriate intake of vitamin A optimizes the body’s defense against microbes. Studies have shown that nutrient deficiencies are responsible for weakened immune responses [64]. The regular intake of vitamin A has been reported to decrease the infectivity and seriousness of many diseases, such as diarrhea, pneumonia, measles, malaria, and AIDS [65]. Vitamin A deficiency decreases bovine coronavirus vaccines’ efficiency and makes the animals more susceptible to infections [66,67]. It has been reported that the infectious bronchitis virus (IBV) infections caused by a strain similar to CoVs were more common in vitamin-A-deficient chickens than in those given vitamin A supplements [68]. The mechanism behind the protective effects of vitamin A against pathogens is its ability to upregulate specific components of innate immunity in healthy cells and fight against viral infection [69,70].

Vitamin A plays a functional and structural role in both the natural and acquired immune systems against several viruses. Supplementation of vitamin A may upregulates the immune system and helps to combat SARS-CoV-2 infections. However, clinical trials to establish the role of vitamin A against SARS-CoV-2 are ongoing (Table 1) [71]. It provides a physical defense by maintaining the structural barrier of mucosal cells in the skin, respiratory tract, and digestive tract. An adequate amount of vitamin A is essential for the optimal functioning of immune cells, including natural killer cells and macrophages, which are components of the innate immunity. Vitamin A helps to ensure the proper functioning of lymphocytes and produce an antibody response against an antigen [47]. It controls the antigen-presenting cells and maintains the balance between Th1 and Th2 lymphocytes [72]. Thus, vitamin A represents a promising alternative to counteract the novel coronavirus’s deleterious effects and prevent lung damage.

### 5.2. Vitamin B

Vitamin B regulates the inflammatory response [73]. B vitamins act as vital cofactors in various cellular reactions and mediate the synthesis of amino acids, the basic structural units of antibodies and cytokines. They play substantial roles in the proliferation and maturation of lymphocytes, which are part of the primary immune response [47]. There are different types of B vitamins (B_2_, B_3_, B_6_, and B_12_), and they are water-soluble and primarily function as coenzymes in various vital processes in the body. Each B vitamin has a unique function and plays a vital role in immunity to combat infections. For example, vitamin B_2_, also known as riboflavin, is involved in cellular energy-yielding metabolic processes [74]. It has been shown that UV light and vitamin B_2_ efficiently decrease the level of MERS-CoV in the human body [75].

Nicotinamide, also known as vitamin B_3_, enhances the destruction of *Staphylococcus aureus* by upregulating specific genes [76]. In silico studies suggest the effect of vitamin B12 has an inhibitory effect on RNA-dependent RNA polymerase activity of the SARS-CoV-2 virus, the main enzyme involved in viral replication [77,78]. Pyridoxal 5′-phosphate, the active form of vitamin B_6_, is involved in protein, carbohydrate, and lipid metabolism and is involved in more than one hundred reactions in the body. Recent research has revealed that the vitamin B_6_-derived bananin (BAN) has inhibitory effects on the SARS-helicase enzyme, which hinders the viral replication process [79]. Vitamins B_6_, B_12_, and B_9_ (folic acid) enhance natural killer cell activity, which provides an important antiviral defense [80]. These findings suggest that B vitamins have the potential to limit the complication related to COVID-19 infection [81]. In view of the studies mentioned above, it is obvious that vitamin B has a crucial role in regulating the inflammatory response, amino acid synthesis, and the proliferation of lymphocytes. 

### 5.3. Vitamin C

Vitamin C (ascorbic acid) is water-soluble, and it plays a significant structural role in the synthesis of collagen, a component of human connective tissues, and is also a powerful antioxidant. Its main function is related to immunity, and it has been shown to protect against various infections, including coronavirus infections [82]. For example, vitamin C can improve the resistance of cultured chick embryos against avian coronavirus infections [83]. This vitamin has antihistamine effects and can relieve flulike symptoms, including runny nose, congestion, sneezing, and inflamed sinuses [84,85]. Human trials have reported a significant decrease in pneumonia incidence when increased doses of vitamin C were given in the diet. This finding suggests that vitamin C has great potential to decrease the vulnerability to lower respiratory tract infections [82]. A clinical trial in the USA reported that intravenous (IV) doses of vitamin C decreased sepsis-induced ARDS death rate. The development of ARDS in patients with COVID-19 is a critical complication that leads to mortality [86].

Recent studies recommended the consumption of vitamin C to control lower respiratory tract infections, and vitamin C supplementation represents one of the most compelling therapeutic interventions for COVID-19 [87,88,89,90]. Vitamin C’s primary function in the immune response against infections is to act as a potent antioxidant. Ascorbic acid is a cofactor for various enzymes involved in biosynthesis and gene regulation processes [39]. Vitamin C mediates the immune response through many cellular functions of the acquired and innate immune systems. Vitamin C provides an epithelial barrier against various pathogenic organisms. It increases the oxidant-scavenging ability of the skin to help to protect against oxidative stress. Vitamin C enhances the chemotactic ability of phagocytic cells, which increases the phagocytosis of invading microbes. It has been shown that vitamin C plays a pivotal role in removing old neutrophils from infection sites and decreasing the potential damage to infected tissues [91]. A randomized clinical trial (NCT04264533) in China is underway in which approximately 140 cases infected with SARS-CoV-2 will be given IV vitamin C (24 g/day for seven days), and it is hoped that the results will be available in September 2020 [92].

### 5.4. Vitamin D

Vitamin D plays dual roles in the body as a nutrient and a hormone. It is produced in response to sunlight and maintains the health of bones. It stimulates the growth and maturation of several cells, including immune cells, and plays a crucial role in immune functions [93]. A significant cause of vitamin D deficiency is sunlight deprivation, particularly in older people who stay indoors [94]. The majority of the COVID-19 cases are middle-aged to older individuals who have inadequate vitamin D levels. Vitamin D deficiency in calves is linked with increased susceptibility to bovine coronavirus infection [95]. Recent research reports have highlighted the role of vitamin D as a potent immunomodulator to combat influenza and COVID-19. It has been suggested that supplementation of vitamin D at an oral dose of 200,000 to 300,000 international units (IU) and other micronutrients for a week can strengthen the immune system against COVID-19 [96,97]. An observational study also demonstrated an inverse relationship between the critical outcome of COVID-19 and serum levels of 25 hydroxyvitamin D (25(OH)D) [98]. However, in contrast, a study from the UK reported no statistical relationship between serum 25(OH) D level and COVID-19 [99].

The physiological role of vitamin D in fighting against infections and diseases has been widely studied. Recently, vitamin D was found to reduce the risk of the common cold and other similar viral infections. It was found that vitamin D helps the immune system in three ways: (i) providing a physical barrier, (ii) strengthening natural immunity, and (iii) strengthening adaptive immunity [100]. 1,25-dihydroxy vitamin D improves the physical barrier that protects against infections. The active hormone encodes proteins that maintain the adherens junctions, tight junctions, and gap junctions in epithelial cells. These proteins strengthen the barrier by holding cells together and improving cell-to-cell communication [101]. It enhances the innate cellular immunity by mediating the release of antimicrobial compounds, including 1,25-dihydroxy vitamin D [5]. Vitamin D also improves the cellular immune system by controlling the “cytokine storm” produced in response to natural immunity. The innate immune response produces inflammatory cytokines against viral infections, including COVID-19. Vitamin D can also decrease the tissue damage induced by the “cytokine storm” [102]. A recent study reported that the crucial molecular virulence mechanisms of COVID-19 infection are based on the interaction of human DPP4/CD26 with spike glycoprotein S1 of SARS-CoV-2 [103]. Optimal vitamin D levels may attenuate this virulent mechanism. In vivo studies have shown that the expression of DPP4/CD26 was significantly reduced after the correction of 25(OH)D deficiency [104]. These findings suggest that vitamin D supplementation is promising for the management of this new viral disease.

### 5.5. Vitamin E

Vitamin E is fat-soluble and is one of the most important vitamins to maintain the immune system [105]. It is a powerful antioxidant and can reduce the levels of oxidative stress in the body by sequestering free radicals [106]. Vitamin E deficiency was found to increase the myocardial injury caused by an RNA viral (coxsackievirus B3) infection in mice, as the viral strains become more virulent under oxidative stress [107]. Likewise, vitamin E deficiency in calves was linked to a high risk of bovine coronavirus infection [95].

Vitamin E is a potent antioxidant that protects the cell membrane from damage by free radicals. It has been stated that vitamin E plays an essential role in enhancing the production of natural killer cells and interleukins. It significantly contributes to the proliferation of lymphocytes and elicits a robust immune reaction against pathogens [25]. These findings support the potential therapeutic use of vitamin E against COVID-19.

## 6. Conclusions and Future Directions

**Figure 2:**
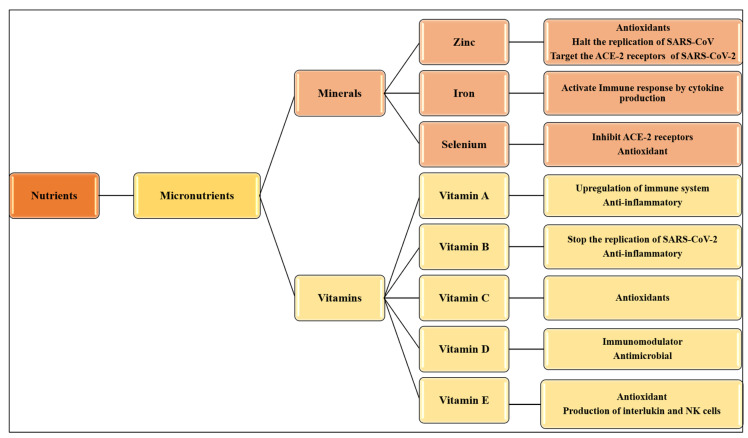
Mechanism of action of micronutrients against CoVs. NK = natural killer; ACE-2 = angiotensin-converting enzyme 2.

This review summarizes the potential nutritional interventions available for various coronaviruses that can be used to combat COVID-19 (Figure 2). Several studies have reported that available nutritional interventions can significantly improve the host immune response against infections by RNA viruses. The supplementation of micronutrients in COVID-19 should not be confused with their administration in cases of their deficiencies. Moreover, nutritional deficiencies weaken the immune response in many model systems as well as in humans. These findings highlight the importance of assessing the nutritional status to identify potential risk factors for viral infections. It is suggested to do a dietary assessment at the time of administration of COVID-19 patients, which may help in the better outcome of treatment. Such compounds proven to be efficient remedies for MERS and SARS may be used to manage COVID-19 and develop new SARS-CoV-2 medications. In contrast to previous research based on a single micronutrient, our analysis focuses on up-to-date awareness of the use of minerals and vitamins and their potential role in the immune system. Moreover, some studies focused on the effect of specific diet patterns, probiotics, and medicinal herbs. Our study is different as we individually discussed the significance of each mineral and vitamin and its potential role in preventing COVID-19 in view of the recently updated scientific information.

This review has several limitations. First, some micronutrients have not yet been shown to have a direct effect on SARS-CoV-2, so the data used in this review were predominantly from studies on related viruses, such as SARS and MERS. Second, most clinical trials to assess these micronutrients are still in progress, and results are not currently available. The results of these clinical trials are thus necessary to determine the direct impact of micronutrients on SARS-CoV-2 and establish safe doses. Additional prospective clinical trials are needed to address these speculations and enhance the knowledge about the association between micronutrients and COVID-19.

## Figures and Tables

**Table 1:** Examples of clinical trials on the use of vitamins against COVID-19 registered by the WHO [8].

Trial ID	Study Design	Sample Size	Settings	Intervention in COVID-19 Patients
ChiCTR2000032400	Cohort	60	China	High dose of vitamin C.
RCT20200401046909N1	Randomized clinical trial	260	Iran	1000 IUs of vitamin D daily for 8 weeks.
IRCT20180520039738N2	Randomized clinical trial	140	Iran	Vitamin A (25,000 IU/day) for 10 days.
DRKS00021214	Randomized clinical trial	1300	Germany	Vitamin B_3_ (nicotinamide) 1000 mg/day for 4 weeks.
EUCTR2020-001602-34-FR	Randomized clinical trial	260	France	A high dose of vitamin D (400,000 IU) versus a standard dose (50,000 IU) once daily for 14 days.
TCTR20200404004	Randomized clinical trial	400	Thailand	Comparison of chloroquine, (10 mg base/kg) and vitamin C (1000 mg).
IRCT20170117032004N3	Randomized clinical trial	30	Iran	Vitamin A (50,000 IU) along with routine treatment for 2 weeks.
CTRI/2020/06/026189	Randomized clinical trial	210	India	Vitamin D (60,000 IU) single-dose and magnesium glycinate (250 mg bi-dose) for 14 days.
IRCT20200319046819N1	Randomized clinical trial	60	Iran	Vitamin A (25,000 IU), Vitamin D (600,000 IU) once during the intervention, Vitamin E (300 IU) twice daily, Vitamin C (500 mg) five times a day, and Vitamin B (Soluvit ampoule) daily.
NCT04335084	Randomized clinical trial	600	USA	Hydroxychloroquine, Vitamin C, D, and Zinc.
NCT04264533	Randomized clinical trial	140	China	Vitamin C (12 g) twice a day for 7 days.

IU, International units.
